# Studies on the Low-Temp Oxidation of Coal Containing Organic Sulfur and the Corresponding Model Compounds

**DOI:** 10.3390/molecules201219843

**Published:** 2015-12-11

**Authors:** Lanjun Zhang, Zenghua Li, Jinhu Li, Yinbo Zhou, Yongliang Yang, Yibo Tang

**Affiliations:** 1 Key Laboratory of Coal Methane and Fire Control, Ministry of Education, China University of Mining and Technology, Xuzhou 221008, China; junjunzhang11@163.com (L.Z.); helloqiqicc@163.com (J.L.); zhouyinbo2011@163.com (Y.Z.); yyliang456@126.com (Y.Y.); 2 School of Safety Engineering, China University of Mining and Technology, Xuzhou 221116, Jiangsu, China; 3 College of Mining Technology, Taiyuan University of Technology, Taiyuan 030024, China; tangyibo11@126.com

**Keywords:** coal, organic sulfur, model compounds, low-temp oxidation, XPS

## Abstract

This paper selects two typical compounds containing organic sulfur as model compounds. Then, by analyzing the chromatograms of gaseous low-temp oxidation products and GC/MS of the extractable matter of the oxidation residue, we summarizing the mechanism of low-temp sulfur model compound oxidation. The results show that between 30 °C to 80 °C, the interaction between diphenyl sulfide and oxygen is mainly one of physical adsorption. After 80 °C, chemical adsorption and chemical reactions begin. The main reaction mechanism in the low-temp oxidation of the model compound diphenyl sulfide is diphenyl sulfide generates diphenyl sulfoxide, and then this sulfoxide is further oxidized to diphenyl sulphone. A small amount of free radicals is generated in the process. The model compound cysteine behaves differently from diphenyl sulfide. The main reaction low-temp oxidation mechanism involves the thiol being oxidized into a disulphide and finally evolving to sulfonic acid, along with SO_2_ being released at 130 °C and also a small amount of free radicals. We also conducted an experiment on coal from Xingcheng using X-ray photoelectron spectroscopy (XPS). The results show that the major forms of organic sulfur in the original coal sample are thiophene and sulfone. Therefore, it can be inferred that there is none or little mercaptan and thiophenol in the original coal. After low-temp oxidation, the form of organic sulfur changes. The sulfide sulfur is oxidized to the sulfoxide, and then the sulfoxide is further oxidized to a sulfone, and these steps can be easily carried out under experimental conditions. What’s more, the results illustrate that oxidation promotes sulfur element enrichment on the surface of coal.

## 1. Introduction

Spontaneous coal combustion is a severe disaster ocurring widely in coal industry. It not only occurs during the coal exploitation process, but also during the processes of coal transport and storage. Meanwhile, it causes great economical losses, as well as safety and environmental issues [1,2,3]. Some scholars believe that deep coal beds have a high sulfur content while shallow coal beds have low sulfur content [4]. As massive scale exploitation and utilization of coal resources continues, the sulfur content in coal is increasing. Sulfur in coal can be classified into two categories: inorganic and organic sulfur. Inorganic sulfur is present in the form of pyrite and sulfate, mainly the former. Often, organic sulfur and the macromolecular structure of coal join together in the form of covalent bonds with complex structure and hard to separate. It mainly includes the form of mercaptans, sulfide, disulphides, thioethers, sulfoxides, sulphones, thiophene, sulfoacids, *etc.* [5,6,7]. It is generally acknowledged that pyrite as the main component of inorganic sulfur exerts an important effect on coal spontaneous combustion. In the presence of water, pyrite reacts with oxygen to form sulfate, H_2_O_2_, and hydroperoxides, and thereby oxidation is initiated [8,9,10,11]. Nearly all coals contain more or less organic sulfur. According to Chinese statistics, low sulfur coal whose total sulfur content is lower than 0.5%, mainly contains organic sulfur. Organic sulfur in high sulfur coal, whose average sulfur content registers 2.76%, accounts for 1.04% of the total amount and 37.7% of the total sulfur [12,13,14]. The organic sulfur content in some superhigh organosulfur coals such as Spanish lignite from Mequinenza and Croatian coal from Raša is over 10%, while, the organic sulfur content in Zelanian coal from Charming Creek (New Zealand) and Indian coal from Tipong is 5% [15]. Because organic sulfur in coal has a complex structure and cannot be separated as it often exists in macromolecular form, its form and content cannot be determined directly so far [16]. People usually use destructive methods to study the forms of organic sulfur in coal. The principle is that HS is produced by pyrolysis and catalytic reduction or SO by oxidation reaction [5]. The analyses mainly involve temperature programmed reduction (TPR) [17], temperature programmed pyrolysis (TPP) [17], temperature programmed oxidation (TPO) [18,19], fast pyrolysis [20] *etc.* Nowadays, some non-destructive technologies such as XANS [21], or XPS [22,23] are widely applied in the determination of sulfur forms in coal. It is noteworthy that the information about surface sulfur in coal samples detected by surface-analysis technologies is not identical to forms of bulk sulfur found [22,24,25].

The oxidation characteristics of organic sulfur and inorganic sulfur in coal during the coal spontaneous combustion process are different. People often ignore the role of organic sulfur during the process of coal spontaneous combustion. From modern chemistry knowledge, knowing that sulfur uses 3p orbital overlap with other atoms to form π bonds, the π bond overlap among 2p and 3p orbitals or between 3p orbitals is smaller, the distance between π electrons and the nucleus is also farther, and C-S bond and S-H bonds are prone to break. Besides, the atomic volume of sulfur is larger, while its electronegativity is smaller, so the valence shell is far from the nucleus, and less influenced by the nucleus. Compared to carbon atoms, sulfur atoms is more easily oxidized [26], so the organic sulfur functional groups in coal are often more active, although the oxidation characteristics of the various forms of organic sulfur differ greatly. For example, the mercapto group in mercaptans has strong reductibility, and it can be instantly oxidized to disulfide with cryogenic air. Under moderate conditions, this reaction can take place and disulfide can be further oxidized to a sulfonic acid [27]. Sulfides such as thioethers also can be oxidized by air to sulfoxides and sulphones. However, –SO_2_ bond connected with aliphatic and aromatic functional groups are prone to C–S bond breakage under low temperature heating conditions, and this releases SO_2_ gas. Under a higher temperature sulfoxides and sulphones with β hydrogens, can be oxidized to sulfinic acids and sulfonic acids [28]. Organic sulfur in thiophene is very stable, even at 500 °C [29]. LaCount *et al.*, reported that the model compound thiophene could release SO_2_ at 450 °C [30] and the reaction can take place slowly with water at 300 °C [31]. From the perspective of dissociation energy, in the structure of R–S–H, the dissociation energy of C–S bond is 20 kcal lower than that of the S–H bond, because the sulfur atom function weakens the bond energy. The C–S bond energy when connected with a phenyl is higher than in the corresponding aliphatic hydrocarbon compound. This is due to the fact that the unpaired electrons and the aromatic structure produce a conjugation effect [32]. Dark *et al.*, claimed that at 20–25 °C the C–S bond easily breaks and produces free radicals, so radicals appear during low-temp oxidation. As the aliphatic carbon atom content increases, the C–S bond dissociation energy decreases significantly [33]. On the contrary, the dissociation energy of an aromatic disulfide S–S bond is lower than an aliphatic disulfide one because it is undermined by the phenyls [32]. The technology for oxidative desulphurization of organic sulfur in coal is based on the characteristic that C–S bonds and S–S bonds are easy to break during low-temp oxidation. At present, this technology has been widely applied in the coal chemical industry. Borah *et al.*, believe that desulphurization of organic sulfur in coal is due to the release of sulfur free radicals under 50 °C, but when temperature reaches 50 °C, along with free radicals, volatile sulfur compounds begins to participate [6]. After oxidation pretreatment, the organic sulfur in coal will be broken down into smaller molecules, thus making further desulphurization easier. They also believe that low-temp oxidation can convert organic sulfur in coal to S=O and –SO_2_ groups [6,30,34]. Gorbaty *et al.*, conducted oxidation experiments on three coal samples with air in 125 °C, the XPS and XANS results showed that aliphatic sulfides are more easily oxidized than aromatic sulfides. The aliphatic sulfides were then converted into sulfoxides, sulfones and sulfonic acids. One important thing is that sulfonic acid is easier to produce in solution than by air oxidation [35,36]. Pietrzak and Grzybek did series of oxidation experiments using different rank coals by using O_2_/Na_2_CO_3_, PPA, and their XPS results showed that the oxidation sequence of the organic sulfur in coal is: sulphide → sulfoxide → sulfone. They also found the sulfur enrichment on the surface of coal during oxidation is probably due to the opening and expansion of coal pores [23,37,38].

All in all, the organic sulfur groups in coal show strong reactivity, and some organic sulfur presents itself as mercaptan, thioether and other groups, which can be oxidized at normal temperature. During oxidation, free radicals can be produced. Due to the easily-oxidized features of organic sulfur, people have conducted plenty of studies on desulphurization that have yielded fruitful results [34,39], but scholars have seldom researched the changes in organic sulfur functional groups during the process of coal low-temp oxidation in air [6], let alone the effect of organic sulfur on the characteristics of spontaneous coal combustion. In light of the above, this paper selects two representative organic sulfur compounds as model compounds. By analyzing the chromatograms of their gaseous low-temp oxidation products and GC/MS scans of the extractable matter in the oxidation residues, we tried to figure out what happens to these sulfur model compounds during low temp-oxidation. On this basis, we conducted an experiment against coal samples containing organic sulfur from Xingcheng, Guizhou Province (China). By adopting XPS technology, we investigated the sulfur form changes in coal samples before and after low-temp oxidation so as to further study the role organic sulfur plays in spontaneous coal combustion.

## 2. The Result and Discussion 

### 2.1. The Result of Experiments with Organic Sulfur Model Compounds

As mentioned previously, the organic sulfur in coal has a complex composition and structure, therefore, nowadays, people still cannot conduct thorough studies on it. However, coal spontaneous combustion is a very complex physicochemical process and its mechanism remains to be unveiled. Therefore, in order to better understand the mechanism of organic sulfur reactions during the spontaneous coal combustion process, this paper selected two representative organic sulfur model compounds and conducted an experimental study on their low-temp oxidation. The use of model compounds to study the complex chemical reaction processes in coal is widely accepted, and it has been applied in the international coal chemistry study field. Model compounds possess some specific functional groups. Therefore, we try to reveal the complex reaction mechanisms of coal molecules by studying the reaction mechanisms of model compounds [40,41,42]. Nowadays, people mainly use organic sulfur model compounds to study the mechanisms of the pyrolysis and desulfurization processes and sulfur’s transformation and transportation. Yan *et al.*, conducted pyrolysis analysis of many sulfur-containing model compounds and concluded the rules of the generation of gases from thioethers, mercaptans, disulphides, and thiophene [43]. Mullens *et al.* conducted an AP-TPR-MS experiment on the model compounds of benzyl thiofuran and dibenzothiophene and found that the corresponding H_2_S release peaks occurred at 545 °C and 690 °C [44], respectively.

#### 2.1.1. The Oxygen Consumption of Model Compounds during Low-Temp Oxidation

**Figure 1:**
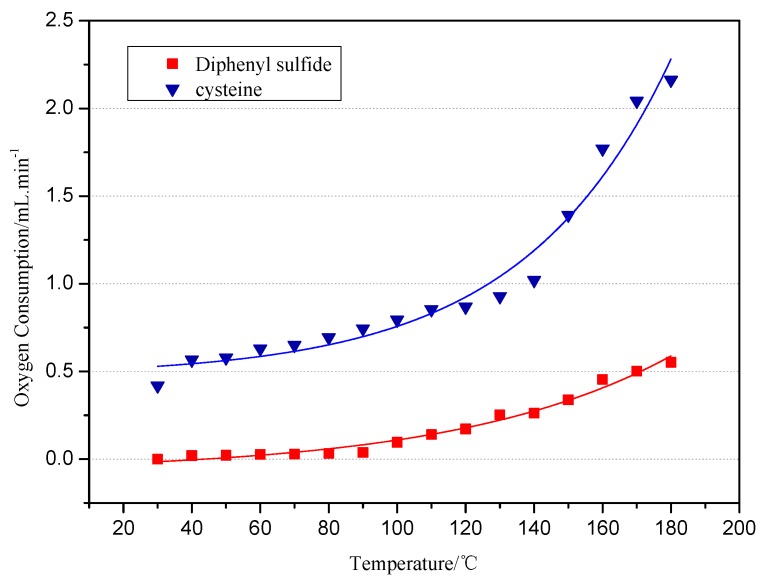
The relationship between the O_2_ consumption and oxidation temperature.

The relationship between the oxygen consumption and temperature during the low-temp oxidation of the diphenyl sulfide and cysteine model compounds is shown in Figure 1. It can be seen from the figure that the oxygen consumptions of both the diphenyl sulfide and cysteine model compound and temperature show an exponential relation. The oxygen consumption of the cysteine model compound is much larger than the oxygen consumption of the diphenyl sulfide model compound, which changes little between 30 °C to 80 °C. When the temperature reaches 90 °C, the oxygen concentration gradually decreases while the oxygen consumption increases. During the low-temp oxidation process, the oxygen consumption increases from 0 mL/min to 0.5 mL/min, which can be explained by the fact that diphenyl sulfide mainly reacts by physical adsorption up to 80 °C. Above 80 °C, it starts to undergo chemisorption and react chemically with oxygen, and the oxygen consumption increases slowly. The cysteine model compound’s oxygen consumption registers 0.418 mL/min at 30 °C, which shows that the active group in cysteine can be oxidized at room temperature. Under 140 °C, the slope curve demonstrates a gradual increase of oxygen consumption, which reflects the slow reaction speed of the active group in cysteine. On the contrary, the oxygen consumption increases sharply above 140 °C, which is attributed to a strong reaction between the active groups and oxygen.

#### 2.1.2. Gaseous Products Generated in the Low-Temp Oxidation Process of Organic Sulfur Model Compounds

CO, CO_2_ and SO_2_ are not detected in the low-temp oxidation process when a gas chromatograph and infrared gas analyzer are used to determine the gaseous product of diphenyl sulfide oxidation.

**Figure 2:**
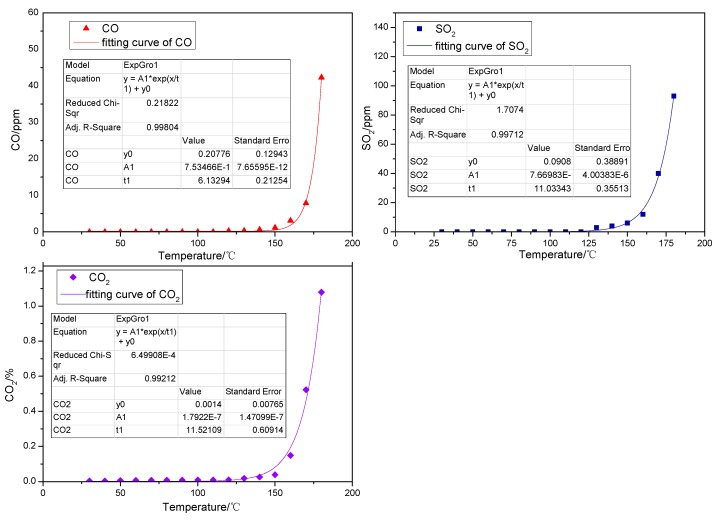
The oxidation product curve of the model compound cysteine.

Yet, as shown in Figure 2, when the above instruments are used to determine the gaseous products of cysteine, the results demonstrate that CO, SO_2_ and CO_2_ are detected during the low-temp oxidation, among which CO_2_ appears first at the lowest temperature and with the highest concentration, while the concentration of both CO and SO_2_ are rather low. According to the relationship between the amount generated and the oxidation temperature of CO, SO_2_ and CO_2_ the gas generation shows an exponential relationship with oxidation temperature, with a correlation index over 0.99. When the temperature is 30 °C, CO_2_ can be detected at a concentration of 0.00336%, which may come from the outside atmosphere. The amount of CO_2_ generated begins to increase gradually when the temperature reaches 50 °C and increases more slowly from 50 °C to 140 °C. Finally, it rises sharply above 140 °C. The concentration of CO_2_ can be up to 1.07% when the temperature reaches 180 °C. The oxidation temperature of the model compound cysteine is 120 °C when CO emerges, while the rate of release slows down a little from 120 °C to 140 °C and then it experiences a larger increase when the temperature is above 140 °C. In general, the concentration of CO during the whole process is not high. SO_2_ appears when the oxidation temperature is 130 °C. The amount of SO_2_ generated increases slightly when the temperature ranges from 130 °C to 140 °C and experiences a larger increase as the temperature increase continues. When the temperature goes up to 180 °C, the concentration of SO_2_ reaches 93 ppm.

#### 2.1.3. GC/MS Analysis Results of Organic Sulfur Model Compounds

**Figure 3:**
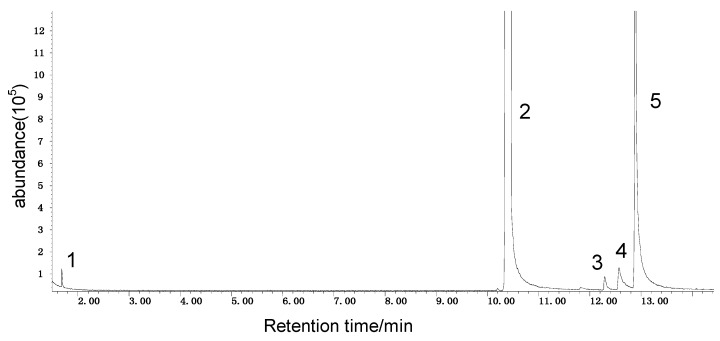
GC-MS of the oxidation products of diphenyl sulfide.

**Figure 4:**
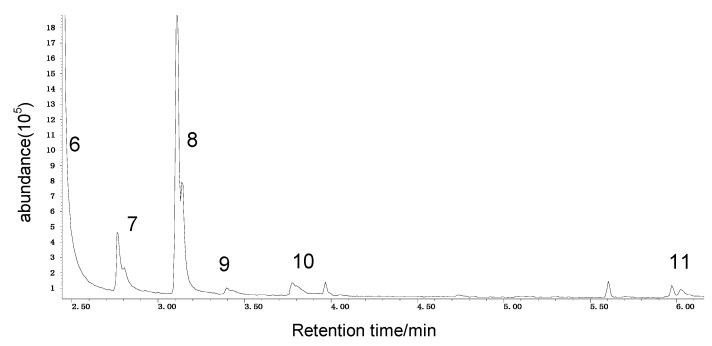
GC-MS of the oxidation products of cysteine.

In this paper, the acetone extract from the low-temp oxidation residues of diphenyl sulfide and cysteine are analyzed using GC/MS. Table 1 shows the name and structure of the peaks marked in Figure 3 and Figure 4.

It can be seen from the GC/MS analysis results of the model compound diphenyl sulfide in Figure 3 that diphenyl sulfide, the compound represented by peak 2, displays the highest content, followed by the content of diphenyl sulfone represented by peak 5. In addition, other substances like diphenyl disulfide, diphenyl sulfoxide and benzene are also detected in the extract. It can be seen from the GC/MS analysis results of the model compound cysteine in Figure 4 that the compound represented by peak 6 contains the highest content of mesityl oxide, while other content ranked in descending order are the compounds represented by peak 8, peak 7, peak 10, peak 9 and peak 11.

**Table 1:** GC/MS-detectable compounds in organic sulfur compound extracts.

Peak Number	Chemical Name	Structural Formula	Peak Number	Chemical Name	Structural Formula
1	benzene	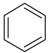	7	diacetone alcohol	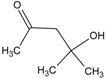
2	diphenyl sulfane	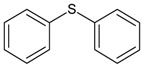	8	5-hexen-2-one	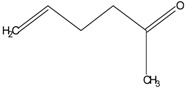
3	diphenyl disulfide	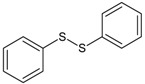	9	4-methoxy-4-methyl-2-pentanone	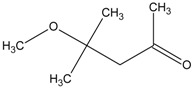
4	diphenyl sulfoxide	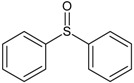	10	4-mercapto-4-methyl-2-pentanone	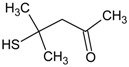
5	diphenyl sulfone	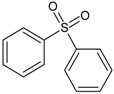	11	3,3,5,5-tetramethyl-1,2,4-trithiolane	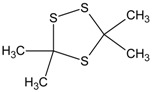
6	mesityl oxide	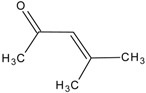	_	________	_____________

#### 2.1.4. The Low-Temp Oxidation of Model Compounds Containing Organic Sulfur

**Figure 5:**
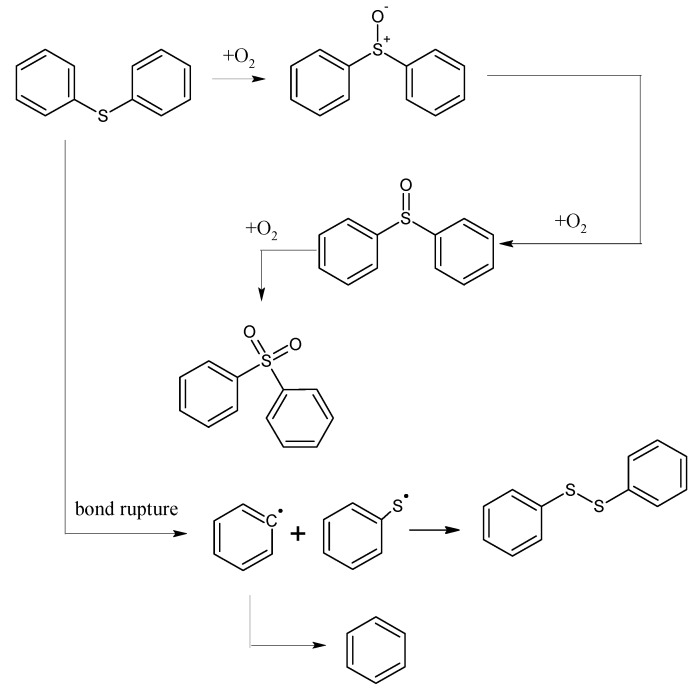
The oxidation pathway of diphenyl sulfide.

The mechanism of the low-temp oxidation process of the model compound diphenyl sulfide can be generally inferred by analyzing the low-temp oxidation products as well as the oxidation residue extract. As shown in Figure 5, in the temperature range of 30 °C~80 °C, the adsorption between diphenyl sulfide and oxygen is mainly physical, and during this time both the oxygen consumption and the oxygen concentration change are small. However, after 80 °C, chemical adsorption and reactions begin to take place and oxygen consumption increases, then diphenyl sulfoxide is generated in the reaction and finally oxidized to diphenyl sulphone. This process is the main reaction mechanism of the low-temp oxidation of diphenyl sulfide. In this process, the C–C bonds in the benzene ring, as well as C–H bonds remain unchanged, with only a series of oxidation reactions of the sulfur atom in diphenyl sulfide ocurring. This is due to the larger nucleophilic reactivity of the sulfur atoms in diphenyl sulfide and diphenyl sulfoxide, which are more easily oxidized compared to the carbon atoms on the benzene ring. Besides, there is also a small amount of homolysis of the C–S bond of diphenyl sulfide, leading to the formation of free radicals. We attribute this to the low dissociation energy of C–S bond in phenyl compounds. Finally, the collision among free radicals generates diphenyl disulfide and benzene. As the C–C bond and C–H bond in the benzene ring remain intact during the low-temp oxidation process of diphenyl sulfide, naturally gases such as CO, CO_2_ and SO_2_ cannot be detected in its products.

**Figure 6:**
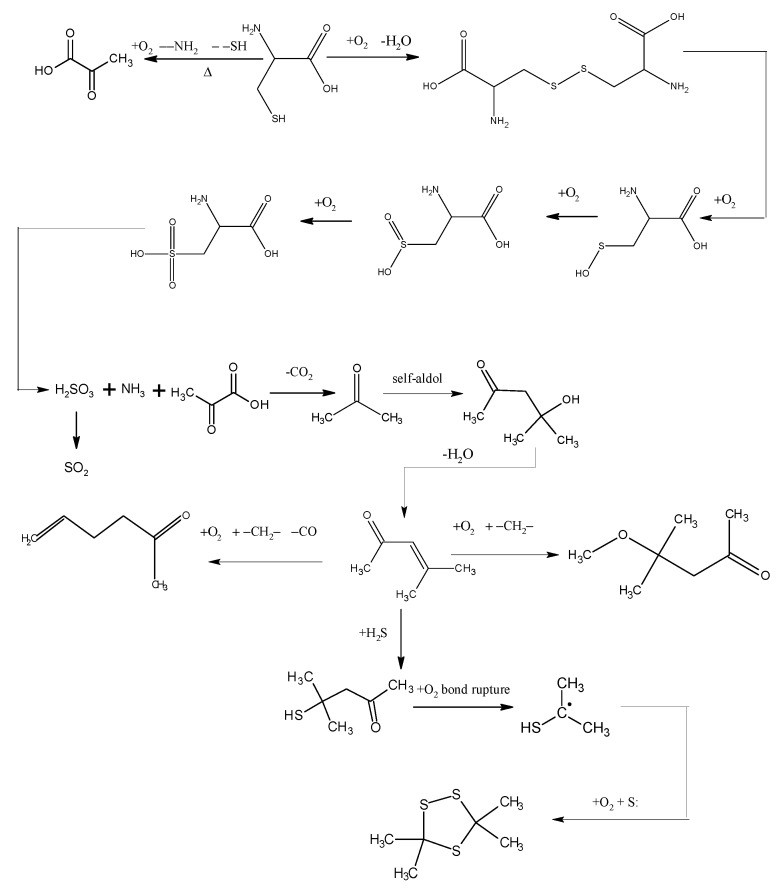
The oxidation pathways of cysteine.

The low-temp oxidation of the model compound cysteine can be generally inferred from analyzing the low-temp oxidation products as well as the oxidation residue extract. As shown in Figure 6, the reducibility of the sulphydryl in cysteine determines that cysteine can be oxidized to disulfide cystine in low-temp air. This reaction can take place when the temperature reaches 30 °C. The model compound cysteine begins to consume oxygen at a rate of 0.418 mL/min. disulfide cystine continues to be oxidized in the air to sulfenic acid, sulfinic acid and sulfonic acid, among which the sulfonic acid generates pyruvic acid, sulfurous acid and ammonia through desulfonation and deamination. Then the sulfurous acid is decomposed into SO_2_ at 130 °C, while the pyruvic acid generates CO_2_ and then acetone after decarboxylation. The condensation of acetone forms diacetone alcohol which generates mesityl oxide after dehydration. The reaction of mesityl oxide with oxygen and methylene generates 5-hexen-2-one, which is accompanied by of a small amount of CO generated at the highest temperature of 120 °C. This reaction also generates 4-methoxy-4-methyl-2-pentanone, the reaction of which with H_2_S generates 4-mercapto-4-methyl-2-pentanone whose chemical bonds break under its reaction with oxygen to generate isopropyl mercaptan free radicals. These isopropyl mercaptan free radicals combine with sulfur free radicals, generating 3,3,5,5-tetramethyl-1,2,4-trithiolane. In general, the large number of active groups in cysteine, such as sulphydryl, amino and carboxy, leads to a more complex oxidation reaction process, with more cross reactions and side reactions, therefore, it can be substantially concluded from the reaction process that the oxidation mechanism of organic sulfur is that mercaptan is oxidized in low-temp air to disulfide, which is further oxidized to sulfinic acid and sulfonic acid. Though desulfonation, the sulfonic acid generates sulfurous acid, which releases SO_2_ by pyrolysis. C–S bond cleavage and sulphydryl detachment can also occur in the mercaptan, leading to the generation of H_2_S gas. S–S bond and C–S bond cleavage in the disulfide can also produce sulfur free radicals.

### 2.2. The XPS Results Before and After the Low-Temp Oxidation of Coal Samples

As has been mentioned, XPS technology, the most effective method of surface elemental analysis so far, is widely employed in the research on the sulfur forms of coal [19,45,46,47,48]. Using this technology, this paper examined the changes in the surface composition of coal samples from Xingcheng, Guizhou Province, before and after low-temp oxidation in order to further understand the characteristics and mechanism of the spontaneous combustion of coal containing organic sulfur.

**Figure 7:**
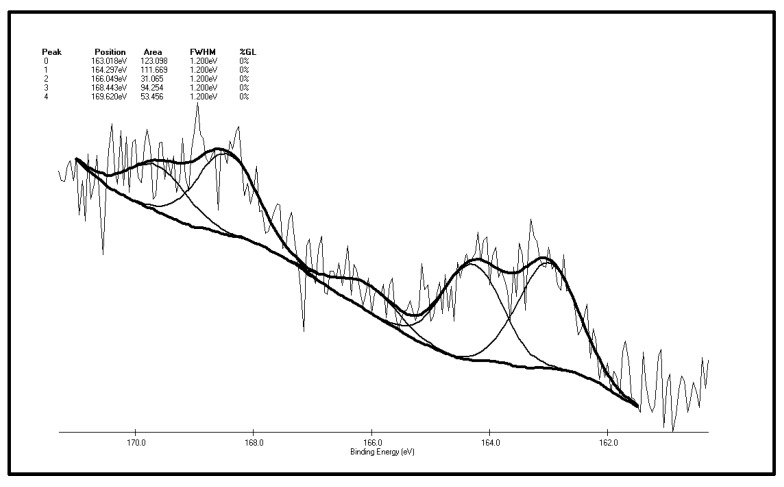
S 2p spectrum of the XF original coal sample.

**Figure 8:**
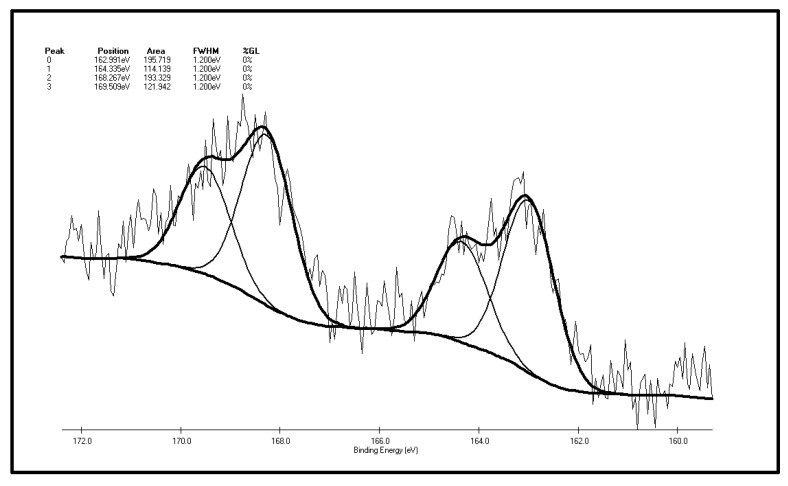
S 2p spectrum of the XF oxidized coal sample.

Figure 7 and Figure 8 are XPS peak separation fitting result charts of element S in the XF sample before and after oxidation. Table 2 and Table 3 are the XPS analysis results of various forms of sulfur in the coal samples before and after oxidation. It can be seen from Figure 7 and Table 2 that there are five S 2p peaks on the surface of the XF original coal samples. As represented by peak 0, the total content of sulfide sulfur and pyrite sulfur is 29.77%. It can be inferred from the higher pyrite content of the coal samples that the coal surface only contains trace amounts of sulfide sulfur. Apart from sulfide sulfur (sulfide sulfur and pyrite sulfur cannot be distinguished due to the low resolution of the experiment) the content of thiophene sulfur, the major component of organic sulfur, is 27%. In addition, there is also sulfone, a small amount of sulfoxide and 17.01% of sulfate detected in the surface of coal samples. Differences exist between the XPS results and the chemical analysis results, which show that the content of pyrite sulfur in coal is 44.77%, while the total content of pyrite sulfur and sulfate is 29.77% in the XPS results. The reason for this phenomenon is that the pyrite in the coal particle surface is easily oxidized to sulfate, leading to the increase of sulfate sulfur content to 17.01% in the XPS results while there is only 0.03% of sulfate in coal in the chemical analysis results. By adding the content of pyritic sulfur, sulfate and sulphide sulfur in the XPS results, it can be obtained that these three forms of sulfur make up 42.7% of the total sulfur ratio, while in chemical analysis results, the sum of pyritic sulfur and sulfate make up 44.38% of the total sulfur ratio. The two results are therefore close, with the ratio in the XPS results being slightly smaller than that of the chemical analysis results. The reasons for this phenomenon are that, on the one hand, as a surface analysis technology, XPS only detects the molecular layer 2–20 in the surface [37], so there is a gap between it and bulk analysis results; on the other hand, the “particle effect” of pyrite might weaken the XPS signal.

**Table 2:** The S 2p spectrum analysis of the XF original coal sample.

Peak	Sulfur Form	Postion	Area	FWHM (Ev)	%GL (%)	*W* (%)
0	Pyritic sulfur + sulphide	163.018	123.098	1.2	0	29.77
1	Thiophene	164.297	111.669	1.2	0	27.00
2	Sulfoxide	166.049	31.065	1.2	0	7.51
3	Sulphone	168.443	94.254	1.2	0	22.79
4	Sulfate	169.620	53.456	1.2	0	12.93

**Table 3:** The S 2p spectrum analysis of the XF oxidized coal sample.

Peak	Sulfur Form	Postion	Area	FWHM (Ev)	%GL (%)	*W* (%)
0	Pyritic sulfur + sulphide	162.991	195.719	1.2	0	31.31
1	Thiophene	164.335	114.139	1.2	0	18.26
2	Sulphone	168.267	193.329	1.2	0	30.93
3	Sulfate	169.509	121.942	1.2	0	19.51

As illustrated by Figure 8 and Table 3 that there are four S 2p peaks on the surface of the XF sample after oxidation. As represented by peak 0, the total content of sulfide sulfur and pyrite sulfur is 31.3%. The content of sulfone, the major component of organic sulfur, is 30.93%, followed by the content of thiophene sulfur, 18.26% and the content of sulfate, 19.51%. Sulfoxide is not detected on the coal surface. The conclusions can be drawn by making a comparison of XPS test results of the XF sample before and after oxidation: the sulfur peak intensity and area of the oxidized sample are higher than the original one, which shows sulfur element enrichment on the surface of coal during oxidation, which is consistent with Graybek’s conclusions. This may be due to the expansion of pores on the oxidized sample surface, which enables sulfur compounds to migrate to the surface [37]. Another reason is the oxidation makes C elements on the surface be consumed via gasification, while less S element is consumed, then the content of coal surface S is relatively increased [22]. In addition, by comparing the XPS peak parameters of sulfur S 2p before and after oxidation, it can be found that: (1) the results show that sulfide and pyrite sulfur increased slightly (from 29.77% to 31.31%), which may result from S element enrichment on the surface; (2) a very interesting phenomenon is the observation of the relative content reduction of thiophenic sulfur (from 27% to 18.26%). This may be due to the large thiophenic sulfur molecules being very stable, and compared with other small molecules find it harder to migrate to the surface of coal; (3) there is no sulfoxide detected on the surface of the coal after oxidation, yet the content of sulfone increases significantly (from 22.79% to 30.93%), indicating that the form of organic sulfur in the coal particle surface changes after low-temp oxidation. That is, sulfide sulfur is oxidized to the sulfoxide, and then the sulfoxide is further oxidized to sulfone. The step where sulfoxide is oxidized to sulfone can be easily carried out under these experimental conditions; (4) sulfate content increased (from 12.93% to 19.15%), suggesting that the pyrites are transformed to sulfate during oxidation on the coal surface; (5) SO_2_ has not been detected throughout the low-temp oxidation process of the XF samples, so it can be inferred by combining the low-temp oxidation mechanism of the above two organic sulfur model compounds that there is no or little mercaptan and thiophenol in the organic sulfur composition of the XF sample, with thiophene and sulfone being its main ingredients. It also contains traces of sulfoxide and sulfide.

## 3. Experimental Section

### 3.1. Low-Temp Oxidation Experimental Facilities

**Figure 9:**
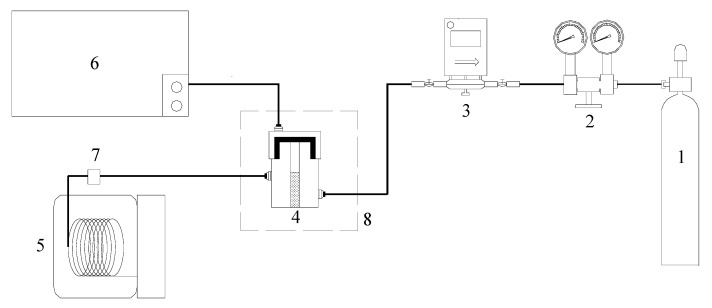
Experimental device of model compound oxidation. 1-Dry Air 2-Pressure Reducing Valve 3-Flow Controller 4-Sample pot 5-Gas Chromatography and Mass spectrometry 6-FUL9790 Gas Chromatography Apparatus & Infrared Gas Analyser 7-Integrated Temperature Sensor 8-Temperature Programmed Furnace.

This paper adopts the low-temp oxidation experimental facilities shown in Figure 9. This system is composed of an air inlet system, coal sample can, an oven for programmed temperature adjustment, temperature control system, gas chromatograph, infrared gas analyzer, and GC/MS. The analysis of the gas product composition of the organic sulfur model compounds under low-temp oxidation conditions has been done using a FUL9790 gas chromatograph and an XLZ-1090 infrared gas analyzer, respectively. This paper uses the FUL9790 gas chromatograph to examine the gas products coming from low-temp oxidation, such as O_2_, CO, CO_2_. We adopted an external reference method to have a quantitative analysis of measured samples. Besides, use the XLZ-1090 infrared gas analyzer to conduct real-time sensing and monitoring of the concentration of SO_2_ under low-temp oxidation conditions. The component analysis of the model compound oxidation residue has been done on an Agilent 6890/5975 GC/MS refering to the standard spectra database (NIST05) to make comparative analyses of the detected compounds.

Agilent 6890/5975 GC/MS test condition: DB-5 capillary chromatographic (column 30 m × 0.25 mm × 0.25 um); Gasification temperature 280 °C; the carrier gas helium; the carrier gas flow rate 1 mL/min; splite ratio 5:1; sample quantity 2 uL; ionization methods EI; Ionization energy: 70 ev; Amu scanning rang 20–650. FUL9790 test condition: environment temperature 5 °C–35 °C; relative humidity ≤85%; carrier gas nitrogen; fuel gas hydrogen; detector temperature 200 °C; thermal conductivity temperature 150 °C, oven temperature 80 °C; the nitrogen pressure is 0.4–0.5 Mpa; the hydrogen pressure is 0.1 Mpa; the air pressure is 0.1 Mpa; TCD current is set to “60”. XLZ-1090 infrared gas analyzer test condition: ambient temperature 0 °C–40 °C; relative humidity ≤90%; the minimum limit (0~1000) × 10^−6^.

### 3.2. Preparation of Laboratory Samples

This paper selects diphenyl sulfide and cysteine as organic sulfur model compounds to perform an experimental study on low-temp oxidation. Table 4 shows the details of the model compounds. These two model compounds separately contain an organic sulfur thioether bond and a mercapto active functional group.

**Table 4:** Model organic sulfur compounds.

Model Compound	Structural Formula	Physical and Chemical Properties
Diphenyl Sulfide	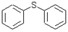	Molecular weight 186.27, colorless or light yellow liquid, malodorous
Cysteine	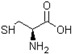	Molecular weight 121.15, colorless crystals

**Figure 10:**
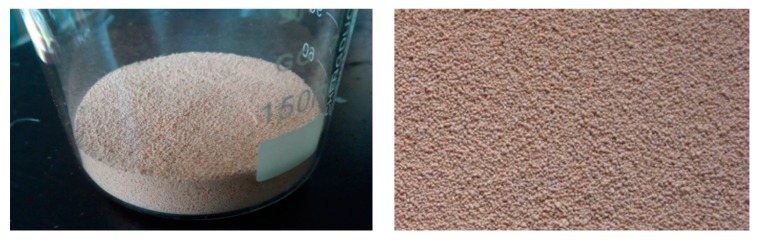
6201-Type Supports.

First, we put organic sulfur model compound on the carrier and distribute it evenly on the surface. The carrier should support the model compounds firmly and have good chemical inertness with good thermal stability which means it does not react with the model compounds and does not take part in any reactions or otherwise disturb the low-temp oxidation of the model compound. After several experiments and analysis, this paper finally selected 6201 support (the particle size is 0.18 mm) as the carrier of model compounds of these experiments. As shown in Figure 10, 6201 support is usually used to fill chromatographic columns and is composed of calcined natural diatomite. Because it has a small amount of ferric oxide, it has pale red color. Its specific surface area is large (specific 4.0 m^2^/g), its average pore size is 1 μm, and its mechanical strength is good.

Using an electronic scale the above model compounds (3 g) were weighed out, acetone (10 g) was added and they were separately placed in a beaker. After mixing them evenly with a glass rod and they are divided into acetone and mixed model compound liquor. 6201 support (30 g) is weighed out and poured into the previous beaker. Then the ingredients are mixed and the mixed liquor is distributed evenly on the surface of the support. The mixture of acetone, model compound and 6201 support in the beaker is placed on a tray and the acetone solution is allowed to completely evaporate, and the model compound will thus be evenly attached on the surface of the 6201 support.

### 3.3. Experimental Procedures

A temperature setting range of 30~180 °C was used in this paper for the low-temp oxidation experiments. The air flow is set to 20 mL/min. Firstly, the 6201 support with the attached organic sulfur model compound is put into the sample can, then the sample can is placed in the programmed temperature adjustment oven, and the air flow is set to 20 mL/min. Next, we turn on the programmed temperature adjustment oven which is heated in 10 °C temperature intervals. When the temperature of the can reaches the setting temperature, we hold that state for 20 min. After that, we analyze the gas composition using the chromatograph and infrared gas analyzer. When the temperature reaches 180 °C, then programmed temperature cycle is stopped and the oven is shut down. After waiting until the sample cools down to room temperature, the sample can is opened and 1 g of 6201 support is removed. Next, we put this 1 g of 6201 support into a breaker, and add 2 g of acetone. After mixing with a glass rod and allowing to stand for 20 min we use a dropper to transfer the extract liquor from the upper layer into a glass bottle, annotating and properly sealing it before using GC/MS to conduct component analysis.

### 3.4. XPS Experimental Samples and Handling

The experimental coal sample is fat coal, Xingcheng Feimei (XF), from Weng’an, Guizhou Province, China. The industry analysis, elemental analysis, and various forms of sulfur analysis of this coal are shown in Table 5 and Table 6. Experimental coal samples (30 g) are taken from a sealed bag and then placed in a vacuum drying oven at 50 °C to dry for 12 h. After they cool down to room temperature, 2 g dried coal sample is ground to 74 μm or less for the XPS test. We put the rest into the sample can and allow it to undergo low-temp oxidation in the experimental apparatus shown in Figure 9 to test for SO_2_ gas emission during the whole process. The test results shows no evidence of SO_2_ during the low-temp oxidation. Afterwards, the 2 g oxidized samples were tested by XPS to conduct a comparison of the XPS results before and after the oxidation.

**Table 5:** The proximate and ultimate analysis of XF coal samples.

Proximate Analysis (wt % as Received)				Ultimate Analysis (wt % Daf)				
M_ad_	A_ad_	Vd_af_	FC_d_	S_t.d_	O_daf_	C_daf_	H_daf_	N_daf_
1.39	16.61	40.17	49.89	2.39	7.11	83.11	5.47	1.43

**Table 6:** The forms of sulfur in XF coal samples.

Forms of Sulfur (wt % db)			
Content	Pyritic	Sulfate	Organic
Absolute (wt %)	1.07	0.03	1.29
Relative (wt %)	44.77	1.26	53.97

XPS measurements are completed on an ESCALAB250 X-ray Photoelectron Spectrometer (Thermo Fisher, Waltham, MA, USA), which uses Al K alpha with power registering 200 W, and the spot size is 900 μm. The pass energy is 20 eV and the base vacuum is 10^−7^ Pa. C 1 s (284.6 eV) is set as the calibration standards. The S 2p XPS spectra obtained receives peak separation fitting by the special software, XPSEAK. As for the specific binding energies of sulfur, 163.1 ± 0.3 eV is attributable to sulfide sulfur and pyrite sulfur, 164.1 ± 0.3 eV to thiophene sulfur, 166.0 ± 0.3 eV to sulfoxide, 168.4 ± 0.3 eV to the sulfone and 169.3 ± 0.3 to sulfates [19,25,35].

## 4. Conclusions

(1) From 30 °C to 80 °C, the adsorption between diphenyl sulfide and oxygen is mainly physical, at which time both the oxygen consumption and the change in the oxygen concentration are small. However, after 80 °C, chemical adsorption and reactions begin to take place and oxygen consumption increases, then diphenyl sulfoxide is generated in the reaction and finally oxidized to diphenyl sulphone. This process is the main reaction mechanism in low-temp oxidation of the model compound diphenyl sulfide. Besides, some free radicals emerge.
(2) The reducibility of the sulphydryl group in cysteine determines that cysteine can be oxidized to cystine, a kind of disulfide, in low-temperature air. This reaction, which is also a common biochemical reaction, can take place under mild conditions. Cystine, a disulfide, continues to be oxidized in air to sulfenic acid, sulfinic acid and sulfonic acid, among which sulfonic acid generates pyruvic acid, sulfurous acid and ammonia through desulfonation and deamination. Then the sulfurous acid is decomposed into SO_2_ at a temperature of 130 °C. Besides, C–S sulphydryl bond cleavage can also occur, leading to the generation of H_2_S gas, while S–S bond and C–S bond cleavage in disulfides may also produce sulfur free radicals.
(3) There are five fitting S 2p peaks on the surface of XF original coal samples. The major inorganic sulfur forms are pyrite and sulfate and the major forms of organic sulfur are thiophene and sulfone. It also contains traces of sulfoxide and sulfide. In accordance with the low-temp oxidation mechanism of the above two model compounds, it can be inferred that there is none or little mercaptan and thiophenol in the coal. Differences exist between the XPS results and chemical analysis results, the reason for which is that the pyrite in the coal particle surface is easily oxidized to sulfate, leading to a much higher content of in the coal particle surface than the bulk phase content of coal samples.
(4) There are four fitting S 2p peaks according to XPS analysis of the oxidized XF sample. The main forms of inorganic sulfur on the surface are still pyrite and sulfate. The major organic sulfur component is sulfone, followed by thiophene sulfur. Sulfoxide is not detected on the coal surface. By comparing the XPS peak parameters of sulfur S 2p before and after oxidation, it can be found that sulfide and pyrite sulfur increased slightly, thiophenic sulfur is reduced, there is no sulfoxide detected on the surface of the coal after oxidation, yet the content of sulfone increases, and the sulfate content also increases too. This phenomenon shows that the form of organic sulfur in the coal particle surface changes after low-temp oxidation. To be more specific, sulfide sulfur is oxidized to sulfoxide, and then this sulfoxide is further oxidized to sulfone. The sulfoxide oxidization step to sulfone can be easily carried out. Sulfur peak intensity and area of the oxidized sample are higher than in the original one, which means there is a sulfur element enrichment on the surface of coal during oxidation.
